# Refugees’ experiences of healthcare in the host country: a scoping review

**DOI:** 10.1186/s12913-017-2731-0

**Published:** 2017-12-08

**Authors:** Elisabeth Mangrio, Katarina Sjögren Forss

**Affiliations:** 1 Department of Care Science, Faculty of Health and Society, Malmö University, Malmö, Sweden; 2 MIM, Malmö Institute for Studies of Migration, Diversity and Welfare, Malmö University, Malmö, Sweden

**Keywords:** Communication, Experiences, Healthcare, Refugees, Scoping review, Support

## Abstract

**Background:**

During the last years, Europe experienced an increase in immigration due to a variety of worldwide wars and conflicts, which in turn resulted in a greater number of physical and mental health issues present among the refugees. These factors place high demands not only on the refugees, but also on healthcare professionals who meet the refugees in different situations. Information about the refugees’ experiences of the healthcare systems in their host countries is urgently needed to improve the quality of healthcare delivered, as well as to provide opportunities for better access. The aim of this scoping review is to compile research about the experiences that the refugees have with the healthcare systems in their host countries.

**Methods:**

This study was conducted as a scoping review and the methodology is derived from Levac et al. and with inspiration from the framework of Arksey & O’Malley. A systematic article search was done in Medline, Cinahl and Psychinfo. A total of 619 articles were found in the search and finally 26 articles met the inclusion criteria and were included.

**Results:**

The results show that communication between healthcare professionals and refugees is important, however, insufficient language knowledge acts as an effective communication barrier. There is a need for more information to be given to the refugees about the reception country’s healthcare system in both oral and written formats, as well as the right to healthcare. Support from healthcare professionals is also important for refugees to have a positive experience with healthcare. In some of the studies included, refugees experienced discrimination due to low proficiency in the language of the host country, and/or because of their race or accent, which shows that culturally appropriate healthcare is needed for them.

**Conclusions:**

Since refugees are suffering from poor mental and physical health and could therefore be at a greater risk of morbidity and mortality in comparison to the rest of the population of the host country, there is an urgent need for improvements in communication, interpretation, support, and deliverance of culturally appropriate healthcare.

## Background

Research suggest that understanding the refugees experience of and access to healthcare are important factors for improving their health [1], as access has been found to be a leading health indicator [2]. Health is an everyday resource and one of the most fundamental conditions for an individual’s potential for development to be fulfilled [3], as well as a key component for an immigrant’s integration into the society and the labour market. However, findings from both Sweden and Canada show a decline in health status among the refugees after settling in their host country [4, 5].

Numerous refugees and asylum seekers undergo both physical and psychological stress in their country of origin, as well as during the transition to and upon arrival in the host country, which can increase their risk of developing mental health problems [6]. Depression and posttraumatic stress disorder (PTSD) are associated with refugees, as it was found that they are more likely to suffer from mental distress in comparison to non-refugees [7]. In a qualitative study done with Iraqi refugee women in the United States, the women expressed and complained about poor physical health, anxiety, stomach pain, psychological discomfort, thyroid disease, chronic disease and other physical and psychological health issues [8]. Another study, based on a survey among newly arrived adult immigrants residing in the US, found that among them chronic health conditions such as high cholesterol, hypertension, overweight/obesity and diabetes were reported. Of the participants, 60% reported to have at least one chronic condition, while 37% reported to have at least two chronic conditions. The prevalence of anxiety, depression and emotional stress was approximately 50% among the participants, and 31% were identified as having PTSD [9]. These findings are in line with an Australian study that screened asylum seekers for mental health status, which shows that 50% of the designated population had mental illness, with around 25% screening positive for PTSD [10]. Various other factors also play a role and may affect the refugees’ health status, including: country of birth, level of ability to communicate in English, community capital, stage of life, and each individual’s balance of protective and risk factors [11].

However, even though they have a higher need for healthcare in comparison to others, refugees face substantial barriers when it comes to healthcare access—barriers [12] that can form as a result of language issues, as well as from cultural and economic aspects [12, 13]. Another factor that also has been found acting as a barrier to healthcare access for refugees is fear, both a fear that visiting a healthcare centre or a General Practitioner (GP) might lead to having their immigration status reported to law enforcement authorities, and a fear that care will be denied [14].

All these factors place high demands not only on the refugees, but also on healthcare professionals who meet the refugees in different situations. Healthcare professionals should understand both the culture of an individual’s country of birth and the immigration experience, but are often unsure about the refugees’ entitlements to healthcare [11], which is a factor that might also influence the refugees’ experience of healthcare. If the experience of seeking care is negative—e.g. feelings of alienation and mistrust—this might prevent the refugees from seeking care in the future [15]. Not seeking care when needed might contribute to the growing health inequality between individuals from different countries of origin that many countries in Europe are facing, as well as the US [16, 17].

During the last years, Europe faced a sharp increase in immigration [18]. The main factors contributing to this increase are both natural and human-generated disasters, including social, economic and political instability [18]. The growing number of refugees presents an immense challenge to the healthcare systems of the countries receiving them [11], and there is a general lack of readiness to handle and understand the healthcare needs of the refugees among healthcare professionals [19]. Information about the refugees’ experiences of the healthcare systems of their host countries is urgently needed to improve the quality of healthcare delivered as well as to provide opportunities for better access [20]. To the best of our knowledge, there has been no earlier study conducted with the same scope. Thus, we undertook a scoping review to compile research about the experiences that refugees have with the healthcare system in the host country.

## Aim

The aim of this scoping review is to compile research about the experiences that refugees have with the healthcare system in their host countries.

## Method

This study was conducted as a scoping review and its methodology is derived from Levac et al. [21]. The intention of the review was to map the literature in the field, rather than to specifically assess the quality of the included studies [22]. We preferred a scoping review to a systematic review since a scoping review can address a broader research question and include a variety of study designs, compared to a systematic review where the focus is on a well-defined question with a limited variety of study designs. This scoping study was inspired by the framework of Arksey & O’Malley [22] and therefore conducted using the following steps: 1. Identifying a research question, 2. Identifying relevant studies, 3. Study selection, 4. Charting the data, 5. Collating, summarizing and reporting results.

### Identifying the research question

How do refugees perceive the healthcare given to them in the host country subsequent to leaving their homeland?

This includes mental healthcare, primary healthcare and hospital care, with the focus being on the perceptions of the adult asylum seekers and refugees. If studies were conducted with both service holders and refugees, the perceptions of the refugees had to be clearly defined in order for them to be considered.

### Identifying relevant studies

Both authors developed the search strategy, as they have previous experience in performing database searches. Literature search was done in Pubmed, Cinahl and Psychinfo, and the following search blocks were built: “refugee”, “healthcare” and “experience”. A search strategy using Medical Subject Headings (MESH) and text words was developed for each keyword and database. See all search terms in Table 1. The literature searches were conducted in March 2017. No limitation was placed on the year of publication, while the language of the publications was limited to English.

**Table 1:** Search terms

Refugees	Primary healthcare	Satisfaction
Asylum seekers	Hospitals	Experience
	Emergency service, Hospital	Lived experience
	Health Services	

### Study selection

**Fig. 1:**
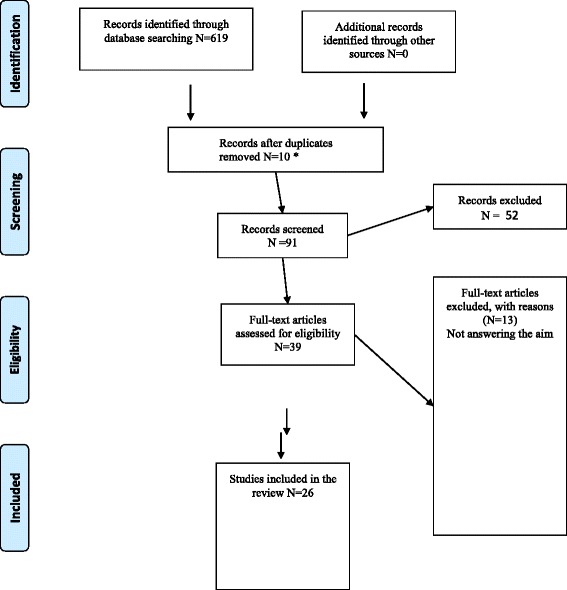
Selection process

The total number of articles found using the search terms in the three databases was 619 (Fig. 1). First, the titles were screened and, if they were in line with the aim, the abstract was read. Using this screening methodology the number of articles was narrowed down to 91. Out of these 91 articles, 10 articles were found to be duplicates and 39 articles appeared to be relevant. These were printed in full, and the authors reached consensus regarding which of them should be included. In this third phase, 13 articles were excluded after scientific appraisal, as they did not address the aim of the study (see Fig. 1). In the end, 26 papers were included and a summary of the eligible studies can be seen in Table 2.

**Table 2:** Summary of included studies

Author and year	Method	Number of participants	Country	Aim	Results
Ahmed A et al. (2008)	Qualitative interviews	10 immigrant new mothers	Canada	To provide insight into attributions of symptoms of depression, their experience with health care and support services, barriers to receive help, and recovery of depression	The majority of the women felt that they could not discuss their feelings of depression with their doctors because they were too rushed, or that the doctors didn’t ask anything about their possible emotional disturbances during the check-ups. In contrast, many of the women had perceived the nurses visiting them after birth to be more helpful and spent enough time with them in order to feel comfortable to raise emotional issues.
Asgary R & Segar N (2011)	Qualitative focus groups interviews	35 asylum seekers and 15 health care providers	United States	To portray the experiences of health care for asylum seekers	The experiences varied between the participants regarding the health care in the US and the asylum seekers had fear of deportation, detention and loss of legal status. They would have liked more information about their health care rights as asylum seekers. They also expressed lack of interpretation and having problems communicating with health professionals.
Bhatia R & Wallace P (2007)	Qualitative interviews	11 adult asylum seekers and refugees	United Kingdom	To determine the views of asylum seekers and refugees regarding the health care experiences.	There were difficulties encountered in many aspects of the health care. Difficulties such as locating practices and experiencing language difficulties and difficulties to get a translator. Some respondents felt discrimination and felt that it had to be dealt with in the whole country and not just in the health care sectors.
Brandon Chen YY et al. (2015)	Qualitative study with focus groups and individual interviews	47 immigrants, refugees and non-status persons living with HIV/AIDS (IRN-PHA)	Canada	To report on the experience for IRN-PHA within mental health care	They reported problem with accessing and utilizing mental health service and this aggravated the stress they experienced and discouraged them for further on accessing support. They experienced stigma and discrimination since they perceived health professionals changing demeanors for example putting on extra pair of gloves. They lacked a sensitivity among health professionals, when handling information about their HIV status. Some had experienced difficulties communicating because of deficiency in English and French.
Cheng IH et al. (2015)	Semi-structured interviews	6 Afghan refugees (3 men and 3 women)	Australia	To analyse the factors influencing Afghan refugees’ access to general practice.	Refugees reported challenges in the language, transport to the practice and appointment waiting times. It was difficult to make appointments because of low proficiency in the English language. Also, they preferred verbal reminders over written reminders.
Donnelly T et al. (2011)	Qualitative interviews	10 women (5 from China, 5 from Sudan)	Canada	To increase understanding of the mental health care experiences of immigrant and refugee women by acquiring information regarding factors that either support or inhibit coping.	The participants reported that they trusted Western biomedicine and its effectiveness in treating mental illness. However, many were unfamiliar with the healthcare system and avoided to seek help as they were not familiar with the ideas of mental health and available treatments for such illness. Limited skills in English, lack of professional interpreters disabled most of the participants from getting access or benefits from mental health counselling services. Lack of information was and where to find information was also reported as well as the need of having written information. Many of the participants had experienced that their health care provider did not spend enough time with them and consequently they felt disappointed and there was a distrust to the health care system.
Fang ML et al. (2015)	Qualitative study with focus groups and individual interviews	35 asylum seekers, refugees and persons without legal status	United Kingdom	To explore health and health care experiences among Somali and Iraqi asylum seekers, refugees and persons without legal status	They reported problems with waiting and that could prolong the process of getting cure for diseases. Another aspect of complaints were lack of knowledge of how to access primary health care services. They also mentioned short consultations and that the doctors were too hastened to encourage full and honest assessments and especially if an interpreter were present. They also mentioned communication barriers for those that had a deficiency in English.
Feldmann CT et al. (2007)	Qualitative interviews	36 refugees participating in 24 interviews	Netherlands	To focus on the relationship between refugee patients and their general practitioners in Netherlands, from the perspective of the refugee patients	Rudeness, anger and impatience were part of several negative episodes. Some of the patients felt that they could not trust or trust their general practitioner, which were seen as an essential element in the health care. Some were very thankful for help given during critical moments at the hospital and others felt that the referral to specialist were too long of a wait.
Herrel N et al. (2004)	Qualitative study with focus groups	14 Somali women	United States	To understand how the Somali women experience pregnancy and childbirth, and improvements that could be done within this health care	Most women spoke highly about the support they had got during labor work, although some women questioned the competence of the nursing staff at the wards. Some felt discriminated on the basis of raceand felt less sensitive care from the staff and others felt discriminated because of not speaking English. They also felt an urgent need from the health care staff to understand the cultural differences of Somali Women. The women would have liked more information about the delivery room experience, pain medication, why prenatal visitsare important, interpretation use at the hospital and how they can expect the staff at hospital to work.
Heydari A et al. (2016)	Qualitative method with semi-structured interviews	19 Afghan refugees	Iran	To explore experiences of Afghan refugees from health service delivery in Iran	They perceived to be discriminated through not being admitted in some hospitals, higher costs and being ignored. They also expressed feelings like being alone and feeling isolated since some Iranians disgusted Afghans and were afraid that they would spread infections. Some participants expressed gratitude for helping them with diagnosing and curing their illnesses.
Lephard E& Haith-Cooper M (2016)	Semi-structured interviews	6 women (4 from sub-Saharan Africa, 2 from Eastern Europe)	United Kingdom	To explore the maternity care experiences of local, pregnant asylum-seeking women.	The women reported pre-booking challenges and also their lack of understanding their entitlement to free health care. Some of the women said that their midwife was a source of support in different ways, however it was also reported that the midwifes did not understand their immigrations status. They also described that they in different situations not were asked or listened to by healthcare personnel (midwifes, GPs, nurses).
Lipson JG et al. (2003)	Qualitative interviews	71 refugees (36 from Bosnia, 35 from former Soviet Union)	United States	To examine health, illness and health care use patterns of all refugees who used Santa Clara County health services during a 29-month period and to explore in more depth the health care experience of refugees from Bosnia and the FSU.	Some differences in the experiences of healthcare were seen among the refugees from Bosnia as compared to those from former Soviet Union. But overall, the participants worried about adequate health care insurance and did not like the long waits for appointment and in emergency room. The health care system was found confusing and the amount of paperwork required for healthcare was a source of distress. Lack of interpreters was a major problem and language barriers i.e., resulted in misunderstanding in directions for taking medications. Health care professionals was seen polite and very professional and the respondents liked the clean and well-equipped facilities.
Murray L et al. (2010)	Semi-structured interviews	10 women	Australia	To uncover first-person descriptions of the birth experiences of African refugee women in Brisbane.	The participants reported no or a little knowledge about the Australian health system and their rights in relation to standard treatment, hospital policies and health education opportunities. They also reported that they were not understood. They did not know that interpreters could be available in the hospital, instead they often used unofficial interpreters. They reported that they did not get any information about ultrasound and they experienced frustration over lack of continuity of care. However, most reported that the midwifes were kind, supportive and helpful.
Neale A et al. (2007)	Questionnaire with open-ended questions and focus groups	98 new arrivals from Iraq (*n* = 35), Afghanistan (*n* = 40) and Iran (*n* = 23). 62 female and 36 male.	Australia	To examine the knowledge of, use of and satisfaction with local primary healthcare services for new arrivals to Australia from Iran, Iraq and Afghanistan.	Confusion and lack of knowledge/information regarding Australian healthcare includingthe public and private systems emerged as a recurring theme. The use of healthcare services yielded significant associations with country of birth groups, type of visa, English language ability and employment status. Gender of the treating doctor and communication were also found as factors influencing the experiences of healthcare. The majority expressed a satisfaction with the care provided, however a number expressed concern with perceived lack of their doctor, and“unease” with the treatment provided. There was also a frustration at the length of time and complicated procedure to be referred to a specialist.
O’Donnell CA et al. (2008)	Qualitative study with focus groups and individual interviews	52 asylum seekers	Scotland	To explore how migrants’ previous knowledge and experience of health care influence their current expectations of the health care in Scotland.	They thought that the health care was good compare to the home country and the concept of free health care for all was welcome. They were used to quick access to doctors visits but didn’t get that every time here in Scotland and that was disappointing for them. Some perceived the GPs to not be specialized and lacked confidence in them. Some thought thatbeing examined by a GP meantto be examined physically and when that was not always done that way, they felt confused with misunderstanding and not understanding the system ofhealth examination here inthe West.
O’Donnell CA et al. (2007)	Qualitative study with focus groups and individual interviews	52 asylum seekers	Scotland	To identify barriers and facilitators to accessing health care, explore health care needs and beliefs among asylum seekers in Scotland	The asylum seekers received written information from health board telling how to register with a GP. But some didn’t get information. Complaints were given regarding long waiting for appointment, not feeling that the GP:s were specialized or lack of continuity. In general the interpretation service were appeared to be well organized, but some lacked interpreters at key points in their hospital stay and interpreters were less common at the dental care compared to the GP.
Omeri A et al. (2006)	Qualitative study with focus groups and individual interviews	38 Afghan refugees	Australia	To explore and describe health and related resettlement issues and barriers that Afghan refugees perceive while seeking health care in Australia.	There was a lack expressed of familiarity with the Australian health care system and the scarcity of Dari speaking interpreters in Australia. Some expressed feelings of discrimination because of accentor lack ofunderstanding. Othersexpressed stereotyping relating to religion and Islamic attire and this were perceived to inhibit access to health care. Complaints existed about gender issues, costsof travel, long waiting times and lack of health related information in Dari. Others were mentioning lack of culturally appropriatehealth promotion in order to help with necessary lifestyle changes.
Owens C et al. (2016)	Qualitative study with a phenomenological framework	12 pregnant refugee women	Australia	To explore the perceptions of care experienced by refugees and migrant women that had participated in an antenatal programme specialized in maternity care	The women appreciated the service given them by the midwifes and doctors at the Health center and appreciated flexible appointment times, but that was not the case regarding appointments at the hospitals. Many of the women lackedfriends and family and could see the midwives as friends andthey appreciated thecontinuity of the same midwife. Interpreters were available but some women wanted to converse in English. The women felt that they had received all necessary information about pregnancy and felt that they could ask questions when necessary.
Penagiota I (2008)	Semi-structured interviews.	26 refugee women.	Greece	To investigate whether refugee women, settled in Greece, receive antenatal care, which elements of antenatal visits are significant to them, which factors influence their attitude towards antenatal care and highlight any barriers that refugees may cope with to access maternity services.	The language barrier affected significantly the participants’ attitudes to antenatal care and also their access to maternity services. Greek maternity services lack interpreting services and written information is in Greek. Language barriers as well as financial barriers were to main reasons for missing appointments. The participants also reported unfamiliarity with the national health system. There was a lack of psychological support as social workers in public hospitals only take care of financial or bureaucratic issues and not issues concerning health. The time factor was also reported to be a barrier that prevented them from using the maternity service as they wanted the staff to have more time to listening to their feelings, discussing different perinatal issues as well as giving advice.
Razavi M et al. (2011)	Qualitative method with semi-structured interviews	Nine refugees with chronic disease and functional impairment	Sweden	To examine the viewpoints of refugees with chronic disease and functional impairment and their contact with health providers	Several participants appreciated having the same physician but some felt that they were sent between different health providers. Some felt that the health care providers showed interest in them as persons butsome felt routine medical examinations were given without commitment. Some informants wished for more information about their disease and treatment.
Redman EA et al. (2010)	A quantitative method with questionnaire	30 asylum seekers	United Kingdom	To describe the self-reported health problems of asylum seekers and their satisfaction with the initial health assessments	Only nine of the informants had received information about the free national health service andthey wished for more informationabout this service. The majority of the informants had received initial health assessment andwere positive about that.
Searight HR (2003)	Qualitative interviews	12 Bosnian immigrants	United States	To understand how Bosnian immigrants experienced and interpreted their interactions with the U.S. health care system.	An universally critical of the US health care system was reported. The participants described several core issues: confusion about insurance coverage, personalized quality of care, access to primary and speciality care; and a perception of U.S health care as bureaucratic.
Shannon P et al. (2012)	Qualitative interviews	50 refugees (32 women, 18 men)	United States	To explore refugees’ perspectives regarding the nature of communication barriers that impede the exploration of symptoms of war trauma in primary care.	Two-thirds of the participants had never been asked by a doctor about the political conflict in their country or the ways they had been affected by it. Many participants did not feel comfortable to start a conversation about their war trauma, but would most likely respond if the doctor initiated a discussion. A majority of the participants reported interest in learning more about the impact of stress and trauma on their health. Language was reported a barrier to communication. Several participants did not appear to define or understand health care as extending to mental health.
Spike EA et al. (2011)	Qualitative study with semi-structured interviews	12 asylum seekers	Australia	To determine whether community based asylum seekers experience difficulties in gaining access to primary health care and to determine the impact of any difficulties described	Some asylum seekers thought it was difficult to be able to see a doctor, since they couldn’t afford the consultation fee and theyexpressed negative experiences of being billed anyway. Some asylum seekers gained access todoctors through charitable services but reported that theiroptions were limited and longwaiting time. Many asylum seekers felt their access to health care was limited by lack of information, particularly when newly arrived.
Valibhoy M et al. (2017)	Qualitative interviews	16 refugees (9 women, 7 men) from Iraq, Iran, Afghanistan, Sudan, DR Congo, Ethiopia, Tanzania, Côte d’Ivoire, Pakistan	Australia	Refugee experiences how they accessed mental health services, their feelings about disclosing personal problems, what promoted and what discouraged engagement with services and practitioners, what assisted them and what they recommended to improve services.	Waiting lists, ineligibility criteria, and continuity of care issues, including referrals from service to service were described as distressing. Cultural responsiveness was very important to participants, and evidently often a challenge for practitioners. Participants wanted practitioners to be ready to learn about and accommodate nuances in ethnic and religious identities.
Wångdahl J et al. (2015)	Cross sectional study	360 Arabic speaking refugees	Sweden	To investigate refugees experiences of communication during their health examination for asylum seekers and the usefulness of the examination and whether health literacy is associated with those experiences	A considerable proportion of the participants experienced that they received little health care information during the examination and the quality of the communication was low. A higher proportion experienced that they were not informed about their rights to health care or where to go if mentally ill. Many of the participants experienced that they did not receive any new knowledge or help during the health examination. Refugees with inadequate health literacy, experienced more often poor quality of communication during health examinations and experienced the health examination less useful. They had also to a lower extent felt that they received enough with health care information.

### Quality appraisal

The aim of this scoping review was to map the literature about refugees’ experiences with healthcare in their host country. As Arksey & O’Malley [22] suggest, a quality appraisal of the included studies in a scoping review is not required. To be included in a scoping review, the only quality criterion a study should have is to be peer reviewed.

### Charting, collating, summarising and reporting the results

After reading the included articles, the data from the articles were interpreted and synthesised. In order to understand the key concepts and sources of evidence found in the literature, a thematic analysis of the material was conducted. All the relevant data that addressed the aim of this scoping review were charted and sorted according to key issues and themes, by extracting findings and key contextual indicators. The two authors, both independently and collaboratively, reviewed the resulting findings. All relevant information about authors, method, number of participants, country, aim and results were divided per article and can be seen in Table 2.

## Results

The results are based on 26 studies from the following countries: Canada (*N* = 3), United States (*N* = 5), United Kingdom (*N* = 4), Australia (*N* = 7), Netherlands (*N* = 1), Sweden (*N* = 2), Iran (N = 1), Scotland (N = 2) and Greece (N = 1). The following themes and headings emerged from the analysis of the studies: communication and information in healthcare, understanding the language, satisfaction with healthcare, dissatisfaction with healthcare, access to healthcare, continuity of care, perceived discrimination, culturally appropriate care and knowledge of healthcare and system.

### Communication and information in healthcare

In a Canadian study by Ahmed [23], the majority of refugee women felt that they could not discuss their feelings of depression with their doctors, either because they were too rushed, or because the doctors did not ask them about possible emotional disturbances during the check-ups. For many women, the post-natal delivery visit from a nurse was the only encounter with a healthcare professional where they could discuss possible feelings of depression. Only one of the women discussed her depression with a psychologist, but did not find it helpful. However, in a study by Herrel [24], which aimed to understand how Somali women experienced pregnancy and childbirth, most of the women in the focus groups felt that they were the key decision-makers during the birth of their child.

In a different Canadian study by Chen et al. [25], some participants felt insufficient or impersonal communication. In an American study by Shannon [26], the authors concluded that two-thirds of the participants were never asked by a doctor about the political conflict in their country, nor how this may have affected them. Many participants did not feel comfortable starting a conversation about their war trauma, but would most likely have responded if the doctor initiated the discussion. Language was also reported to be a communication barrier. According to Wångdahl et al. [27], refugees with inadequate health literacy, which could be described as lesser knowledge about health, experienced poor quality of communication during health examinations more often, and consequently found the health examinations less useful.

According to several studies, more information needs to be provided about the participants’ healthcare rights as asylum seekers [28], about their disease [29] and about the delivery room experience—e.g. pain medication, why prenatal visits are important, using interpretation services at the hospital and what they can expect from the hospital staff [24]. In a study by Murray [30], the participants reported that they did not get any information about the ultrasound. According to a Canadian study by Donnelly et al. [31], there was a lack of information about how to cope with mental illness and its related problems. In a Scottish study by O’Donnell et al. [32], the results showed that the asylum seekers were supposed to receive written information from the health board telling them how to register with a general practitioner (GP), but some did not get this information. According to Owens [33], the informants felt that they received all the necessary information about pregnancy and that they could ask questions when necessary. Redman et al. [34], showed that only nine out of the 30 informants had received information about the free National Health Service and that they wished for even more information about this service. In the Swedish study by Wångdahl et al. [27], the results showed that a considerable portion of the informants felt that they received little healthcare information during the examination, and that the quality of communication was low. At least 30% of the informants did not understand what they were being told. A higher portion were not informed at all about their rights to healthcare, nor where to go if mentally ill. Refugees with inadequate health literacy had to a lower extent felt that they received enough with healthcare information and experienced not receiving any help with health problems.

### Understanding the language

According to the study by Asgary [28], the informants pointed out that there was a lack of interpretation, that they experienced difficulties finding interpreters and were having problems communicating with health professionals. The findings from Bhatia [35] confirm those from Asgary [26], as the informants in this study also experienced language barrier difficulties and difficulties in obtaining a translator. Sometimes appointments with doctors had to be rescheduled because no translator showed up. It was only those refugees that were accompanied by a friend, relative or refugee agency staff that could undergo a trouble-free registration process. In the study by Chen [25], some of the refugees experienced difficulties communicating because they lacked English and French language skills. Cheng [36] found that it was difficult for refugees to make appointments because of low proficiency in the English language, and that because of the language problem they preferred verbal reminders over written reminders. In a study by Fang [37], language barriers for those that had a deficiency in English were also mentioned, with many participants noting that interpreters were either not available or, if they were, there was a problem with confidentiality. This issue arose from the fact that the majority of the interpreters were from the same community as the refugees themselves, and therefore they were afraid of personal information being disclosed among the community. According to Herrel [24], all of the women had used interpreters during their delivery, but many thought that the interpreters were not competent in medical terminology and several suggested that sometimes the patients had more knowledge of medical terms in comparison to the interpreters. In a study by Lipson [38], lack of interpreters presented a major problem, and language barriers resulted in misunderstanding of instructions for proper usage of medications. In the study by Murray [30], the participants also reported that they were not understood. They did not know that interpreters could be provided in the hospital; instead, they often used unofficial interpreters. According to O’Donnell et al. [32], the results showed that in general the interpretation services appeared to be well organized and reliable within primary healthcare, but some participants lacked interpreters during key moments of their hospital stay. According to a study by Omeri et al. [39], there was a scarcity of Dari-speaking interpreters in Australia. In the study by Owens et al. [33], interpreters were available, but some women wanted to converse in English. None of the women with limited or no knowledge of English had an interpreter at birth, but their husbands could speak English. According to a study by Penagiota [40], Greek maternity services lack interpreting services and written information is in Greek. Language barriers, as well as financial barriers, were the main reasons for missing appointments. In the Canadian study by Donnelly et al. [31], limited skills in English and lack of professional interpreters disabled most of the participants from getting access or benefits from mental health counselling services.

### Satisfaction with healthcare

In the study by Ahmed et al. [23], many of the women perceived the nurses visiting them after birth to be helpful. The nurses spent enough time with them in order for the women to feel comfortable enough to raise emotional issues. In the study by Murray [30], most women also reported that the midwives were kind, supportive and helpful. In the American study by Herrel et al. [24], the findings showed that most women spoke highly about the support they received during labour. According to a study by Lephard et al. [41], some of the women said that their midwife was a source of support in different ways and in the study by Owens et al. [33], the informants appreciated feeling understood by the midwife, doctor and staff at the health centre. Many lacked friends and family, and could see the midwives as friends. They saw the midwife as the only practical help that they would get during pregnancy. According to Lipson [38], healthcare professionals were seen as polite and very professional, and the respondents liked the clean and well-equipped facilities. According to a study by Neale et al. [42], gender of the treating doctor and communication were also factors influencing the refugees’ experiences of healthcare and the majority expressed satisfaction with the care provided. In the Swedish study by Razavi et al. [29], some participants felt that the healthcare providers showed interest in them as persons by engaging into a small conversation about daily life with them, for instance, and that was seen as a sign of commitment, knowledge and skill and according to Feldmann et al. [43], some were very grateful for help provided during critical moments at the hospital. In a Scottish study by O’Donnell CA et al. [44], the results showed that the refugees thought healthcare was good when compared to the country of origin, and that the concept of free healthcare for all was welcome. Two issues appeared to build asylum seekers confidence in the GP’s: 1) seeing the same doctor each time they attended the surgery, and 2) the feeling that the doctor respected them. For some of the respondents it was important that the GP not only listened to them medically, but also tried to understand their situation as asylum seekers. And similar results could be seen in the study by O’Donell et al. [32], where most reported positive experiences regarding the care provided by their GP’s and in the study by Redman et al. [34], the majority of the informants received initial health assessments and were positive about that. Heydari et al. [45] reported that some participants expressed gratitude towards the healthcare personnel for helping them diagnose and cure their illnesses, for their honest work and impartial care, and also for realising what their problems were and cooperating with them.

### Dissatisfaction with healthcare

According to the study by Panagiota et al. [40], there was a lack of psychological support, as social workers in public hospitals only take care of financial or bureaucratic issues and not issues concerning health. The time factor was also reported to be a barrier that prevented them from using the maternity service as they wanted the staff to have more time to listening to their feelings, discussing different perinatal issues as well as giving advice [40]. Another aspect that was mentioned was the attitude of the people staffing the service centres, where some women felt very discouraged by the staff that did not seem interested in them [23]. In the Canadian study by Donnelly et al. [31], many of the participants had experienced that their health care provider did not spend enough time with them and consequently they felt disappointed and there was a distrust to the health care system. In the American study by Asgary et al. [28], the authors could see that experiences varied among the participants regarding healthcare in the US, and that the asylum seekers had a fear of deportation, detention and loss of legal status. Some felt that the doctors did not pay much attention to them, but thought that that was because they did not have documents and could not therefore complain. According to the study by Fang et al. [37], the informants also mentioned short consultations with the doctors being too hasty to encourage full and honest assessments, especially if an interpreter was present. In a Dutch study by Feldmann et al. [43], the results showed that rudeness, anger and impatience were part of several negative episodes. Anger about a request to see a female practitioner for a gynaecological problem, impatience towards a young and worried mother bringing her child in with a cold, and unfriendly remarks about Dutch language skills towards a recently arrived woman. Some of the informants felt that they could not trust their GP, which is considered an essential element of healthcare and others felt that the referral to a specialist was too long of a wait. It was also reported that the midwives did not understand their immigration status [41]. The informants also described that in various different situations they were not asked nor listened to by healthcare personnel (midwifes, GPs, nurses) [41]. According to O’Donnell et al. [32], the refugees were not feeling that the GPs were specialized e.g. hoping for a referral to secondary care but instead receiving a prescription or lack of continuity of care e.g. seeing a different doctor each time they attended the surgery [32] or they felt that the care had not met their expectations [32]. In the study by Omeri et al. [39], complaints arose about gender issues, costs of travel, long waiting times and lack of health-related information in the Dari language. In an American study by Searight [46], the authors found that a universal criticism of the US healthcare system was reported. The participants described several core issues: 1) confusion about insurance coverage, 2) personalized quality of care, 3) access to primary and specialty care and 4) a perception of US healthcare system as bureaucratic. In the study by Razavi [29], some participants felt routine medical examinations were given without commitment. In Several of the studies, the participants expressed lack of confidence in the GP’s [42, 44] and in the nurses [24, 42].

### Access to healthcare

According to the study by Asgary et al. [28], some participants experienced difficulties with payments for the doctor’s visits and were praying that they would not get sick. In the Australian study by Spike et al. [47], the results showed that some asylum seekers thought it was difficult to see a doctor, since they could not afford the consultation fee, and they reported negative experiences of being billed anyway. Some gained access to doctors through charitable services, but reported that their options were limited and required long waiting times. This resulted in physical suffering, stress, anxiety and, in some cases, serious health illnesses. In the American study by Lipson [38], the authors noted differences in the experiences of healthcare among the refugees from Bosnia when compared to those from the former Soviet Union. The differences in the experiences are based on the historical circumstances of their refugee status and economic factors in their home countries and health care systems. Overall, however, the participants worried equally about adequate healthcare insurance, and did not like the long waiting times for appointments and in the emergency room. In the study by Cheng et al. [36], the refugees also reported challenges with appointment waiting times and the transport to the clinic. Long waiting times were also confirmed as an issue in the studies by O’Donnell et al. [32] and Fang et al. [37], where the authors concluded that the refugees’ problems with waiting times could prolong the process of obtaining treatment for their diseases [37]. Bhatia [35], also noted that the participants had difficulties in locating clinics.

Lack of knowledge about how to access primary healthcare services was another aspect that caused grievance. In the study by Fang et al. [37], the informants also lacked familiarity with the UK health system, and had limited knowledge of the different health services that were available, as well as of the processes and procedures for accessing health services. According to the study by Neale [42], the use of healthcare services yielded significant associations with country of birth groups, visa types, English language ability and employment status. There was also a frustration among the participants about the time-consuming and complicated nature of the specialist referral procedure. According to O’Donnell et al. [44], the participants were used to having quick access for doctors’ visits, but were disappointed when they did not get it for every visit in Scotland. In the study by Omeri et al. [39], some experienced stereotyping related to religion and Islamic attire, and this was perceived to inhibit access to healthcare. In the Australian study by Owens et al. [33], the results showed that the women appreciated the services provided to them by the midwives and doctors at the health centre and the flexible appointment times, but that was not the case with hospital appointments. In the Greek study by Penagiota [40], the authors found that the language barrier affected the participants’ attitudes to antenatal care significantly, as well as their access to maternity services.

Many asylum seekers felt their access to healthcare was limited due to a lack of information, particularly upon first arrival. In an Australian study by Valibhoy et al. [48], waiting lists, ineligibility criteria and referrals from service to service, were described as distressing. In the Canadian study by Chen et al. [25], a problem with accessing and utilizing mental health services was reported, which aggravated the stress that the participants experienced and discouraged them from accessing support later on.

### Continuity of care

In the study by Chen et al. [25], some participants felt that sometimes the health professionals failed to follow up on them as promised. One woman mentioned that she had to wait for 2 months for a counsellor appointment and described the waiting period as tiring and life-threatening. In the study by Razavi [29], the results showed that several participants appreciated having the same physician, but others felt that they were sent back and forth between different health providers. Therefore, they felt that they did not have a permanent care provider in charge of them who would take on the overall responsibility. This was confirmed in the study by Murray [30], where the refugees experienced frustration over the lack of continuity of care, as well as in the study by Bhatia [35], where some of the refugees wished for more continuity with both the same GP and the same translator in order to develop trust. At the same time, in the study by Owens [33], the refugees appreciated the continuity that having the same midwife during the entire pregnancy provided. However in the Australian study by Valibhoy et al. [48], the refugees felt stressed about the lack of continuity of care within the mental health care.

### Perceived discrimination

In the study by Chen et al. [25], the participants experienced stigma and discrimination when they noticed that the health professionals were changing demeanours—putting on an extra pair of gloves, for example. They also noted a lack of sensitivity among the health professionals when handling information about their HIV status, and felt confronted with discrimination that stemmed from their HIV and immigration status merged together. In the study by Herrel et al. [24], many of the women made negative comments about the care that they received from the nursing staff at the wards. Some felt discriminated against on the basis of race and felt that the staff was less sensitive when providing care, while others felt discriminated against due to not speaking English. In the Iranian study by Heydari et al. [45], the results showed that the participants perceived to be discriminated against when they were not admitted to some hospitals, as well as when they faced higher costs and were being ignored. They also expressed that they felt alone and isolated, since some Afghans were disgusted by Iranians and were afraid that they would spread infections. This view on Afghan people was not just concerning the ones recently arriving, but also the ones that had been living in Iran for many years. In the study by Omeri et al. [39], some participants expressed feelings of discrimination due to having an accent or lack of understanding. According to Bhatia [35], some respondents experienced discrimination and felt that it had to be dealt with in the whole country, not just within the healthcare sectors, in order for the refugees to feel comfortable in the healthcare system.

### Culturally appropriate care

In the study by Fang et al. [37], the authors highlighted that beyond addressing the language issue there was also a need for a more culturally competent system, and that there was a need to address cultural differences concerning symptomologies, diagnoses and medical terminology. In the study by Herrel [24], the authors also felt that there was an urgent need for the healthcare staff to understand the cultural differences of Somali women. This study also suggested that all members of a healthcare team should receive cultural sensitivity training that includes topics such as Somali culture, traditions, values and Somali patient expectations. According to O’Donnell et al. [44], some participants thought that being examined by a GP meant to be examined physically, so when that was not always the case they felt confused, not understanding the system of health examinations here in the West. In the study by Omeri et al. [39], lack of culturally appropriate health promotion to help with necessary lifestyle changes was mentioned. It was suggested that more assistance should be given through information in the Dari and Farsi languages, and they wanted the services given to reflect Islamic teachings. In the study by Valibhoy et al. [48], cultural responsiveness was very important to participants, and evidently it often presented a challenge for practitioners. Participants wanted practitioners to be ready to learn about and accommodate the nuances in ethnic and religious identities.

### Knowledge about healthcare and system

In the English study by Lephard & Haith Cooper [41], the authors found that women reported pre-booking challenges and also lacked understanding of their entitlement to free healthcare. According to the study by Lipson [38], the healthcare system was confusing to many, and the amount of paperwork required for healthcare purposes was a source of distress. In the Australian study by Murray [30], the authors could see that the informants reported little or no knowledge about the Australian health system and their rights in relation to standard treatment, hospital policies and health education opportunities. In two other studies from Australia [39, 42], the authors noted that confusion and lack of knowledge/information regarding Australian healthcare, including the public and private systems, emerged as a recurring theme. According to the study by Penagiota [40], the informants also reported unfamiliarity with the national health system. In the Canadian study by Donnelly et al. [31] many were unfamiliar with the healthcare system and avoided to seek help as they were not familiar with the ideas of mental health and available treatments for such illness. In the study by Shannon [26], a majority of the participants reported interest in learning more about the impact of stress and trauma on their health. Several participants, however, did not appear to define or understand healthcare as extending to mental health. In the study by Razavi [29], some informants wished for more information about their disease and treatment strategy, while patients expressed poor understanding of the diagnosis and why a specific category of care provider should be consulted. According to Wångdahl [27], many of the participants did not receive any new information or help during the health examinations, and felt that they received new health knowledge during the visits in low extent.

## Discussion

The results of this scoping review show that communication between the health professionals and the refugees is important, but that insufficient language knowledge acts as a communication barrier. There is a need for the refugees to be provided with more information about the healthcare system, in both oral and written formats, as well as with the right to healthcare in the reception countries. Support given by the health professionals is of great importance for a positive encounter with healthcare. Waiting times, economy and transportation are perceived to be barriers to healthcare access, and continuity of care is also of importance. In some of the included studies, a certain portion of refugees reports that there is discrimination due to low proficiency in the national language, and/or because of race or accent, and that there is a need for culturally appropriate healthcare improvements. Furthermore, knowledge regarding the healthcare system of the host country is generally poor and more information about it needs to be provided to the refugees.

In several of the included studies, the authors conclude that the refugees and asylum seekers need to be given more information about their rights to healthcare, as well as about the healthcare system itself [24, 27–30, 34], while in only two of the included studies the participants report receipt of such information [32, 33]. In the Swedish study by Wångdahl [27], 30% of the participants did not fully understand what they were being told. This could affect the refugees’ access to healthcare, and reduce their autonomy in informed decision-making concerning their own healthcare [27]. The need for refugees to have proper and effective communication with the healthcare segment is urgent, since they are at a greater risk of suffering from poor physical and mental health [8–10], with increased morbidity and mortality rates in comparison to the rest of the host country population [49]. Vulnerable populations, such as the refugees, often have significant difficulties with health literacy and are therefore challenged by intercultural communication barriers when accessing and making sense of relevant health information. Therefore, these consumers are often misinformed about healthcare services, disease prevention practices and early detection guidelines, which could lead to serious health errors and health problems [49].

A number of the included studies express difficulties concerning interpretation—with finding an interpreter, the confidentiality of using an interpreter, as well as with the quality of the interpretation [24, 28, 30, 32, 35, 37–40]. Ngo-Metzger et al. [50] found that if the consumers of care did not have interpreters present at the healthcare appointment, this meant that they received significantly less health education. Furthermore, even the patients that used an interpreter rated their care provider fair or poor, in comparison to those that knew the native language. Ngo-Metzger et al. [50] concluded that for patients with another native language it is of great importance to have an interpreter, but that having an interpreter may not completely ameliorate the language barrier that exist and may interfere with the patient-provider relationship.

Some of the included studies further show there is a need for a more culturally appropriate healthcare for refugees and asylum seekers [24, 37, 39, 44]. These studies found that culturally appropriate care is needed for several reasons: 1) to address cultural differences concerning symptomologies, diagnoses and medical terminology [37], 2) to increase the understanding of cultural differences [24], 3) to increase the understanding of the health examination system in the West [44] and 4) to develop culturally appropriate health promotion in order to help with necessary lifestyle changes [39]. Cultural competence could be viewed as a critical factor and an essential component for providing relevant, effective and culturally responsive healthcare services to an increasingly diverse population worldwide. Even though cultural competence alone is not sufficient for reducing all health and healthcare disparities, it nevertheless remains one of the most significant tools when it comes to addressing health disparities in society [51]. However, cultural awareness within healthcare requires genuine efforts to be made for cultural barriers to be understood—through engagement in the community, as well as through working closely with members of the community in order to address these barriers [52]. If cultural safety principles are incorporated into healthcare practices and research, then health structures may shift to resist dominant views of culturally and socially heterogeneous groups [37].

In this scoping review a total of 26 studies were included representing 10 different countries around the world, with a higher representation from the countries of Canada, United States, Australia and the United Kingdom. It is important that similar studies are conducted in more countries, to obtain a better picture of the extent to which healthcare is appropriate and positively evaluated by the refugees worldwide. The next step would be to acquire more specific knowledge about the experiences of the different refugee ethnic groups, for then healthcare systems could tailor their services even further to address the unique health needs of these specific groups, and would thus be able to provide genuine culturally appropriate healthcare [53].

In a number of the studies included in this review, the refugees perceived healthcare professionals to be supportive towards them [23, 24, 29, 30, 33, 38, 41, 42, 45], although in a few of the studies the participants were less content and satisfied with these encounters [28, 37, 40, 42 43]. Since this group of people tends to suffer more from poor physical and mental health, and since they also struggle with resettlement challenges [54], it is of importance for health professionals to support them when they come for a wide variety of reasons into healthcare settings. In order to ensure quality healthcare services, it is important that the GPs themselves are supported through professional development in refugee health, so that they are enabled to provide appropriate and suitable care for a group of people with complex health issues [55].

### Strengths and limitations

This review has a broad scope and attempts to draw a picture of the experiences refugees have with the healthcare sector in their host country. One limitation is that the scope may not be broad enough, since only scientific papers were included. A complete search has not been done and, as this is a scoping review, we did not conduct a quality assessment of the reviewed studies. Thus, the results should be interpreted with some caution. Additional search terms, search blocks, or inclusion and exclusion criteria could have been used, although the range of the study and its results would then have been different. Systematic searches have been carried out in three databases that are primarily used for medical and nursing research [56]. Some duplicates were found in the three databases, which suggests that the search breadth was wide enough. Previous work experience of the authors within the field of both immigration and healthcare, as well as experience of performing systemic searches, strengthen this study. The fact that the inclusion and exclusion criteria have been firmly set from the beginning and were followed closely and strictly throughout the data collection process could be seen as an asset to this study. Only English language sources were retrieved and reviewed. Literature on this topic may very well have been produced in other languages, and could therefore be missing in this present review. However, there is very little literature that is not written in English cited in the papers reviewed thus far, which indicates that the most important sources for this topic may be available primarily in English.

## Conclusions

The aim of this review was to compile research about the experiences that refugees have with healthcare sectors in their host countries. The review concluded that insufficient language knowledge is perceived as a communication barrier, that the refugees are lacking information both regarding the healthcare system itself and their right to access it in the host countries. Support given by the health professionals is of great importance for a positive encounter with healthcare, and there is a need for improvement when it comes to provision of culturally appropriate healthcare. Since refugees are at a greater risk of suffering from poor mental and physical health, and therefore have higher morbidity and mortality rates in comparison to the rest of the host country population, there is an urgent need for improvements to be made in communication, interpretation, support and deliverance of culturally appropriate healthcare.

## Abbreviations

EM: Elisabeth Mangrio
GP: General practitioner
KSF: Katarina Sjögren Forss
PTSD: Posttraumatic stress disorder
